# Potential Molecular Mechanism of TNF Superfamily-Related Genes in Glioblastoma Multiforme Based on Transcriptome and Epigenome

**DOI:** 10.3389/fneur.2021.576382

**Published:** 2021-02-11

**Authors:** Hui Xie, Ce Yuan, Jin-jiang Li, Zhao-yang Li, Wei-cheng Lu

**Affiliations:** ^1^Department of Histology and Embryology, College of Basic Medicine, Shenyang Medical College, Shenyang, China; ^2^Graduate Program in Bioinformatics and Computational Biology, University of Minnesota, Minneapolis, MN, United States; ^3^Department of Neurosurgery, General Hospital of Northern Theater Command, Shenyang, China; ^4^Department of Laboratory Animal Center, China Medical University, Shenyang, China; ^5^Department of Neurosurgery, First Affiliated Hospital of China Medical University, Shenyang, China

**Keywords:** glioblastoma multiforme, differentially expressed genes, tumor necrosis factor superfamily genes, DNA methylation, survival analysis

## Abstract

**Objective:** This study aimed to investigate the molecular mechanism of tumor necrosis factor (TNF) superfamily-related genes and potential therapeutic drugs for glioblastoma multiforme (GBM) patients based on transcriptome and epigenome.

**Methods:** Gene expression data, corresponding clinical data, and methylation data of GBM samples and normal samples in the TCGA-GBM and GTEx datasets were downloaded. The TNF-related genes were obtained, respectively, from two groups in the TCGA dataset. Then, the TNF-related differentially expressed genes (DEGs) were investigated between two groups, followed by enrichment analysis. Moreover, TNF superfamily-related gene expression and upstream methylation regulation were investigated to explore candidate genes and the prognostic model. Finally, the protein expression level of candidate genes was performed, followed by drug prediction analysis.

**Results:** A total of 41 DEGs including 4 ligands, 18 receptors, and 19 downstream signaling molecules were revealed between two groups. These DEGs were mainly enriched in pathways like TNF signaling and functions like response to TNF. A total of 5 methylation site-regulated prognosis-related genes including TNF Receptor Superfamily Member (TNFRSF) 12A, TNFRSF11B, and CD40 were explored. The prognosis model constructed by 5 genes showed a well-prediction effect on the current dataset and verification dataset. Finally, drug prediction analysis showed that zoledronic acid (ZA)-TNFRSF11B was the unique drug–gene relation in both two databases.

**Conclusion:** Methylation-driven gene TNFRSF12A might participate in the development of GBM via response to the TNF biological process and TNF signaling pathway and significantly associated with prognosis. ZA that targets TNFRSF11B expression might be a potential effective drug for clinical treatment of GBM.

## Introduction

Glioblastoma multiforme (GBM) is the most aggressive cancer that represents 15% of all brain tumors (1). The most common length of survival following diagnosis is 12 to 15 months, with fewer than 3% to 5% of people surviving longer than 5 years (2). Typically, surgery after chemotherapy and radiation therapy are commonly used for the treatment of GBM (3). However, the cancer usually recurs due to poor effect of existing drugs or treatment strategies on the diffusive, infiltrative, and metastatic of GBM (4).

Further understanding of the molecular mechanisms involved in the development of GBM may contribute to the development of new therapies and strategies (5). Recent observations after immunotherapies with cytokines suggest an immunological and even clinical response from immunotherapies (6). Actually, members of the tumor necrosis factor (TNF) superfamily (TNFSF) and TNF receptor superfamily (TNFRSF) have crucial roles in both innate and adaptive immunity (7). A previous study shows that tumor necrosis factor (TNF) and the associated receptor superfamily play important roles in the development of GBM (8). Some TNFs such as TNF-α are upregulated in GBM cells, which further play an important role in GBM progression (9). TNFRSF19 is upregulated in advanced glial tumors and promotes glioblastoma cell invasion (10). Furthermore, DNA methylation plays an important role in gene expression regulation during the development of tumor (11). Abnormal epigenetic modification can lead to tumors, genetic disorders, inflammation, aging, and neuropsychiatric abnormalities (12). A previous study shows that epigenetic therapy with inhibitors of histone methylation suppresses DNA damage signaling and increases glioma cell radiosensitivity (13). Methylation profiling can be used to identify different groups of GBM according to their tumorigenesis (14). However, due to the lack of integrated analysis of epigenomic and transcriptome data, the specific role of DNA methylation sites and TNF-related gene expression changes in GBM progress, as well as potential effective drugs associated with these genes, are still unclear.

In this study, the gene expression data, clinical information, and methylation data of GBM tumor samples and normal tissue samples in TCGA-GBM dataset and GTEx dataset were downloaded. The differentially expressed genes (DEGs) were explored between tumor and normal samples, followed by function and pathway enrichment. Then, the expression of TNF superfamily-related genes and upstream methylation regulation mechanism was investigated to explore candidate genes and prognostic models. Finally, the protein expression level of candidate genes was performed, followed by drug prediction analysis. This study hoped to investigate the molecular mechanism of TNF superfamily-related genes and potential therapeutic drugs for GBM patients.

## Materials and Methods

### Data Acquisition

A total of 96 TNF superfamily-related genes including 18 TNF superfamily (TNFSF), 29 TNF receptor superfamily (TNFRSF), and 49 related downstream signal genes were enrolled for the current analysis based on literature review (15). The RNA-seq data including corresponding methylation and clinical phenotype data of GBM samples in TCGA and GTEx were downloaded from the University of California Santa Cruz (UCSC, http://xena.ucsc.edu/) Genome Browser database (16). A total of 166 GBM samples obtained from the TCGA-GBM dataset were enrolled as the tumor group. Meanwhile, 5 normal paracancerous tissue samples from the TCGA-GBM dataset and 105 normal brain cortex samples from the GTEx dataset were combined as the normal group. Moreover, after being annotated by hg38 gene annotation information (gencode.v23.annotation.gene.probemap) based on the GENCODE database (17) and having filtered low-level expression genes (not expressed in half of all samples), a total of 76 TNF superfamily-related genes (Supplementary Table 1) including 7 TNFSF, 21 TNFRSF, and 48 downstream signal molecules were extracted for subsequent analysis.

The methylation data of GBM patients was obtained by the correspondence between the chip methylation spectrum site and the symbol annotation conversion file downloaded from UCSC-Xena. Furthermore, clinical data including age, race, gender, radiotherapy and chemotherapy information, new tumor information, OS status, and OS time of each patient in the downloaded data were obtained using TCGAbiolinks in R (18).

### Differentially Expressed Analysis

The linear regression and empirical Bayesian methods (19, 20) in limma package of R (21) were used to explore DEGs between the tumor group and normal group based on the TNF-related 76 gene expression matrix. The Benjamin & Hochberg method was used for multiple-test correction. The adj*P* < 0.05 and | log2 fold change (FC)| > 1 were selected as the thresholds for DEG screening. Then, the volcano plots and clustering heat map were constructed using ggplot2 (version: 3.2.1) (22) and using pheatmap (version: 1.0.12) (23), respectively.

### Enrichment Analysis of DEGs

Gene ontology-biological process (GO-BP), GO-cellular component (GO-CC), and GO-molecular function (GO-MF) (24), and Kyoto Encyclopedia of Genes and Genomes (KEGG) pathway (25) enrichment analyses of TNF-related DEGs were performed using the Metascape software (parameter: min overlap = 3; *P*-value cutoff = 0.01; min enrichment = 1.5) (26). *P* < 0.01 was considered as the cutoff value of significant enrichment results. Moreover, clustering analysis was conducted according to the similarity of genes enriched in each term (similarity of > 0.3). The most statistically significant term (*P*-value minimum) in each cluster was selected to define this cluster. Then, the top 20 clusters based on the *P*-value were visualized by a histogram. Furthermore, to further explore the relationship between terms, the interaction network diagram of terms in the top 20 clusters was constructed (inclusion criteria: terms with the most significant P in each cluster; <15 terms in each cluster; no more than 250 terms in total; similarity > 0.3). Finally, the network was constructed by Cytoscape software (version: 3.4.0) (27).

### Correlation Between Methylation Level and DEG Expression

Based on the correspondence between methylation sites and genes, all methylation sites corresponding to the differential genes were extracted, and the Pearson correlation coefficient (r) between each site and its corresponding gene expression level was calculated and tested for significance. Finally, the *P* < 0.05 and *r* < −0.4 were selected as cutoff values to screen the methylation-related genes.

### Prognosis Analysis Based on DEGs and Methylation Sites

The expression value of DEGs in each sample and the associated patient clinical survival information were used for the DEG prognosis analysis. Univariate Cox regression analysis was used to analyze the associations between DEGs and prognosis, and the DEGs with *P* < 0.05 were considered as the prognosis-related genes. Meanwhile, the methylation value of methylation sites that correspond to prognosis-related genes and the associated patient clinical survival information were used for the methylation site prognosis analysis. The univariate Cox regression analysis was used to analyze the relationship between each methylation site and prognosis. *P* < 0.05 was considered as the cutoff value for candidate prognostic methylation sites.

### Prognostic Model Construction and Verification

The prognostic gene that significantly negatively correlated with the prognostic methylation site was considered as the methylation site-regulated prognosis-related gene. Then, these genes were screened by multivariate Cox expression regression. Based on the prognostic correlation coefficient β and the combination of the expression values of selected genes, the risk score calculation model was defined as

$$\begin{array}{l} Risk\;score\;=\;\beta_{gene1}\times expr_{gene1}\;+\;\beta_{gene2}\times expr_{gene2}\;\\ +\;\ldots +\;\beta_{geneN}\times expr_{geneN} \end{array}$$

The corresponding risk score of each sample was calculated, and the samples were divided into high-risk group or low-risk group based on the median risk score. To reveal the relationship between high/low-risk group and prognosis, the Kaplan–Meier (KM) (28) survival curve and heat map were used to assess the survival time distribution and gene expression value of the two groups. To validate the risk model, the expression profile data of WHO IV grade samples, including GBM, rGBM, and sGBM (DataSet ID: mRNAseq_325) (29, 30), were downloaded from the CGGA database (http://www.cgga.org.cn/download.jsp). Clinical information such as gender, age, chemoradiation information, OS status, and OS time in these data were further enrolled in this study.

### Independence Analysis of Prognostic Models

To investigate whether the prognostic model could be independent of other clinical variables (including age, gender, etc.), univariate Cox regression analysis was performed based on independent variables including high/low-risk groups, age, and gender. Then, the factors with P < 0.05 were enrolled for the multivariate Cox regression analysis. All investigation was performed based on TCGA and CGGA datasets, followed by visualization with forest plots.

### Protein Expression Level Verification

The Human Protein Atlas (HPA) is a database used to study protein expression in different human tissues and organs from RNA and protein levels by transcriptomics and proteomic techniques (31). In order to verify the difference in protein level of the key candidate genes, the HPA database was used to reveal the protein immunohistochemical level of key genes in cortex of normal people and GBM patients.

### Drug–Gene Interaction Prediction

The drugs targeted by diseases-related genes were screened using the Drug–Gene Interaction database (DGIdb, version: 2.0) (32). Based on the drug–target gene relations, the drug–target gene interaction network was constructed by using online database STITCH (parameters: species = homo; medium confidence score = 0.4) (http://stitch.embl.de/) (33).

## Results

### DEGs Between Normal Group and Tumor Group

**Figure 1:**
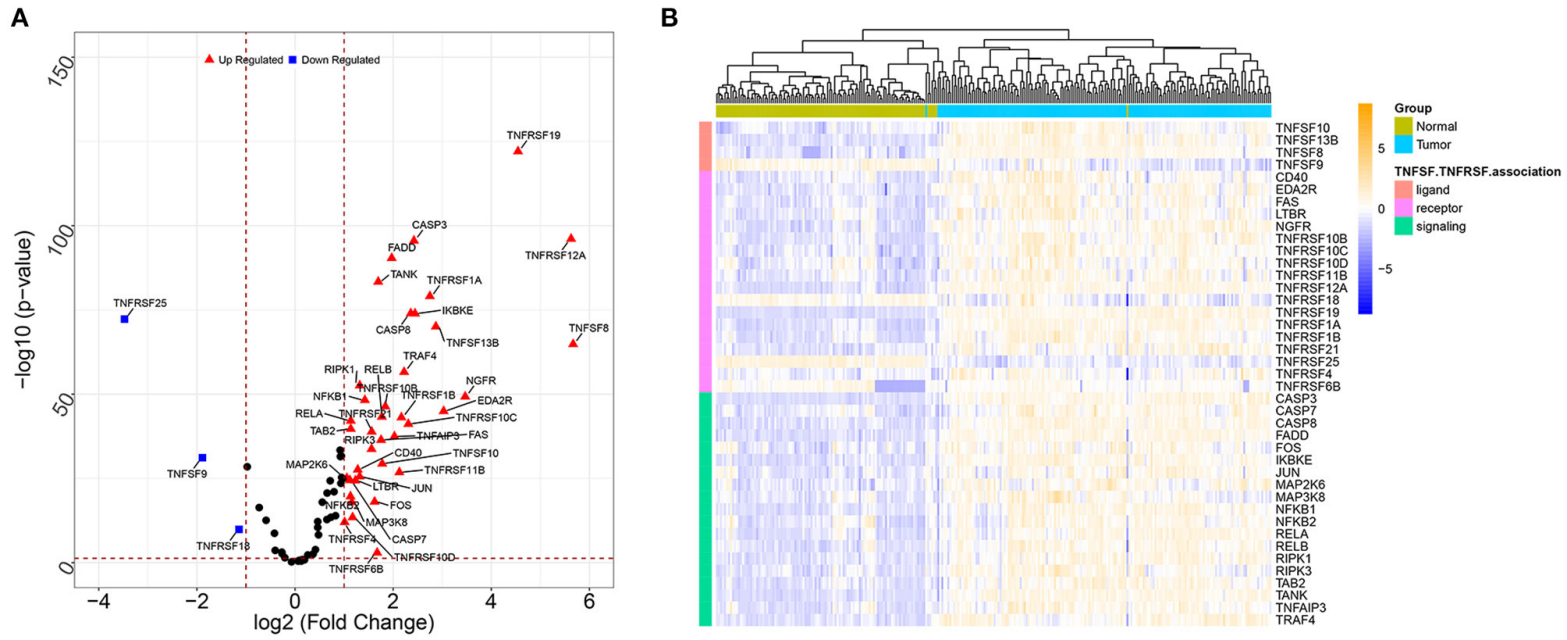
The volcano plots and heat map for DEGs between the tumor sample and normal sample. **(A)** The volcano plots of DEGs; the X-axis represents the value of log2 fold change, while the Y-axis represents the value of –log10; red triangles represent the upregulated genes, blue squares represent the downregulated genes, and black nodes represent genes with no significant difference. **(B)** The heat map for DEGs; colors from blue to yellow indicated low to high representation values. The colored blocks at the top represent samples, of which brilliant blue represents tumor samples and pea green represents normal samples; the colored blocks at the left represent DEGs.

A total of 41 TNF-related DEGs including 4 TNFSFs, 18 TNFRSFs, and 19 downstream signal molecules were identified between the tumor and normal groups. The volcano plot showed that the upregulated genes and downregulated genes were significantly separated (Figure 1A). The heat map showed that the samples could be obviously distinguished by DEGs (Figure 1B).

### Significant GO Function and KEGG Pathways Enriched by DEGs

**Figure 2:**
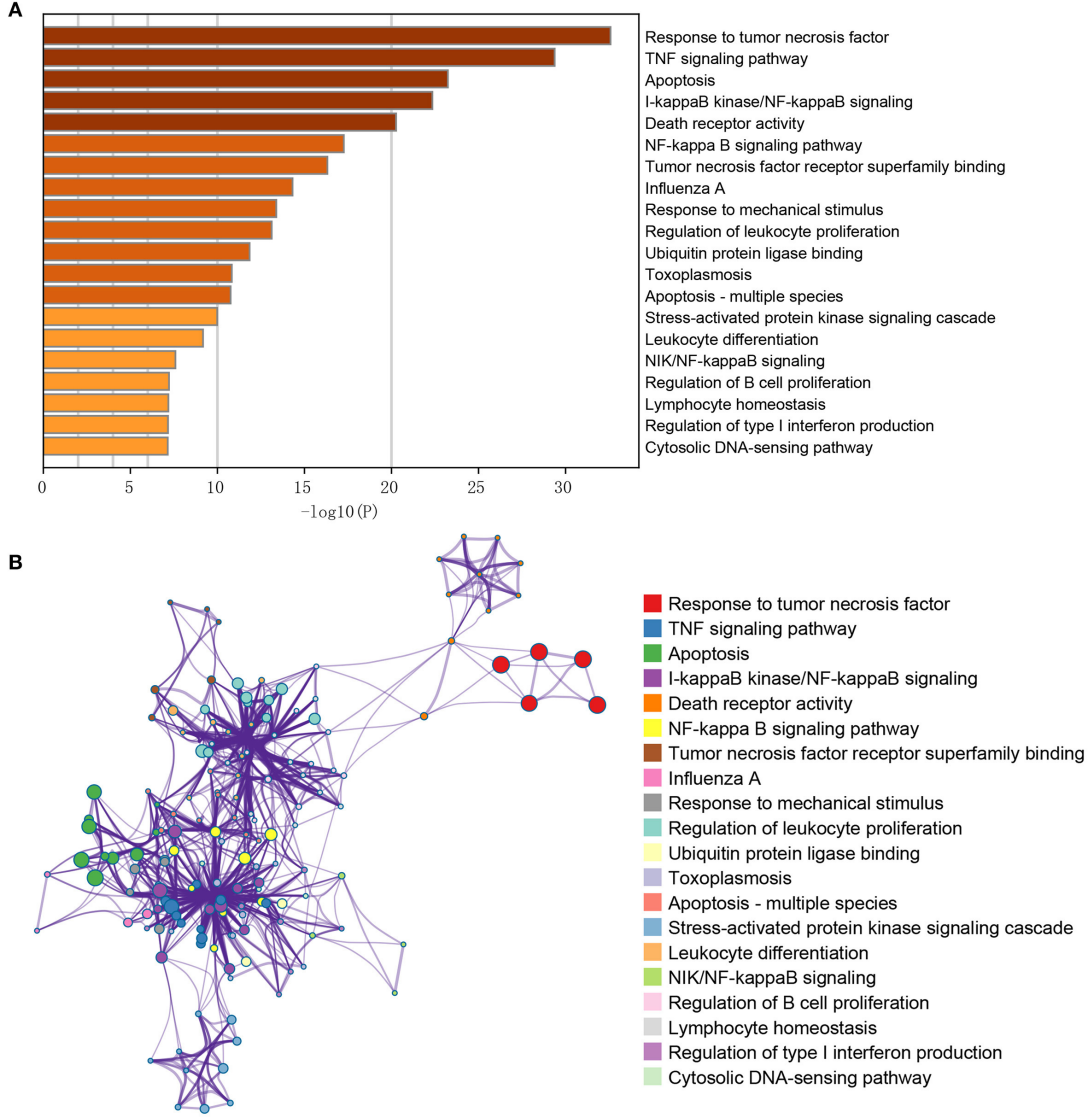
GO/KEGG pathway enrichment cluster interaction analysis of the differentially expressed genes. **(A)** The X-axis represents the gene ratio (–log10); the Y-axis represents the different items of functions or pathways. **(B)** The interactive network among terms; different node colors indicated different clusters, and lines indicated gene similarities among terms.

The obtained DEGs were significantly enriched in 103 GO-BP, 1 GO-CC, 19 GO-MF, and 32 KEGG pathways in the current functional enrichment analysis. These GO terms and KEGG pathways were clustered into different categories based on the similarity cluster analysis. The top 20 cluster is shown in Figure 2A. The results showed that these DEGs were mainly enriched in GO functions like response to tumor necrosis factor (GO: 0034612), death receptor activity (GO: 0005035), and tumor necrosis factor receptor superfamily binding (GO: 0032813). Meanwhile, these DEGs were mainly enriched in KEGG pathways including the TNF signaling pathway (hsa04668), apoptosis (hsa04210), and NF-kappa B signaling pathway (sha04064) (Figure 2A). Moreover, the investigation of the interaction among terms in each cluster is shown in Figure 2B. Each node represents a term, and the nodes with the same color represent the terms in the same cluster. As expected, the terms with more similarity were always clustered in a functional module, while the terms in different clusters showed less interactions.

### The Methylation Site–DEG Interaction and Prognostic Gene Investigation

Based on the corresponding relationship between methylation sites and DEGs, all methylation sites corresponding to DEGs were extracted. A total of 504 methylation sites were obtained. The Pearson correlation coefficient (r) between each methylation site and the expression level of its corresponding gene was calculated to screen the methylation-related genes. A total of 39 methylation sites corresponding to 16 DEGs were obtained with the cutoff value of *P* < 0.05 and *r* < −0.4.

Furthermore, a total of 14 prognosis-related genes were obtained. The associations between methylation sites that correspond to prognosis-related genes and prognosis were calculated, and 74 methylation sites that correspond to 25 prognosis-related genes were obtained to be associated with prognosis (Supplementary Table 2).

### Prognosis Model Constructed by Methylation-Driven Genes

**Figure 3:**
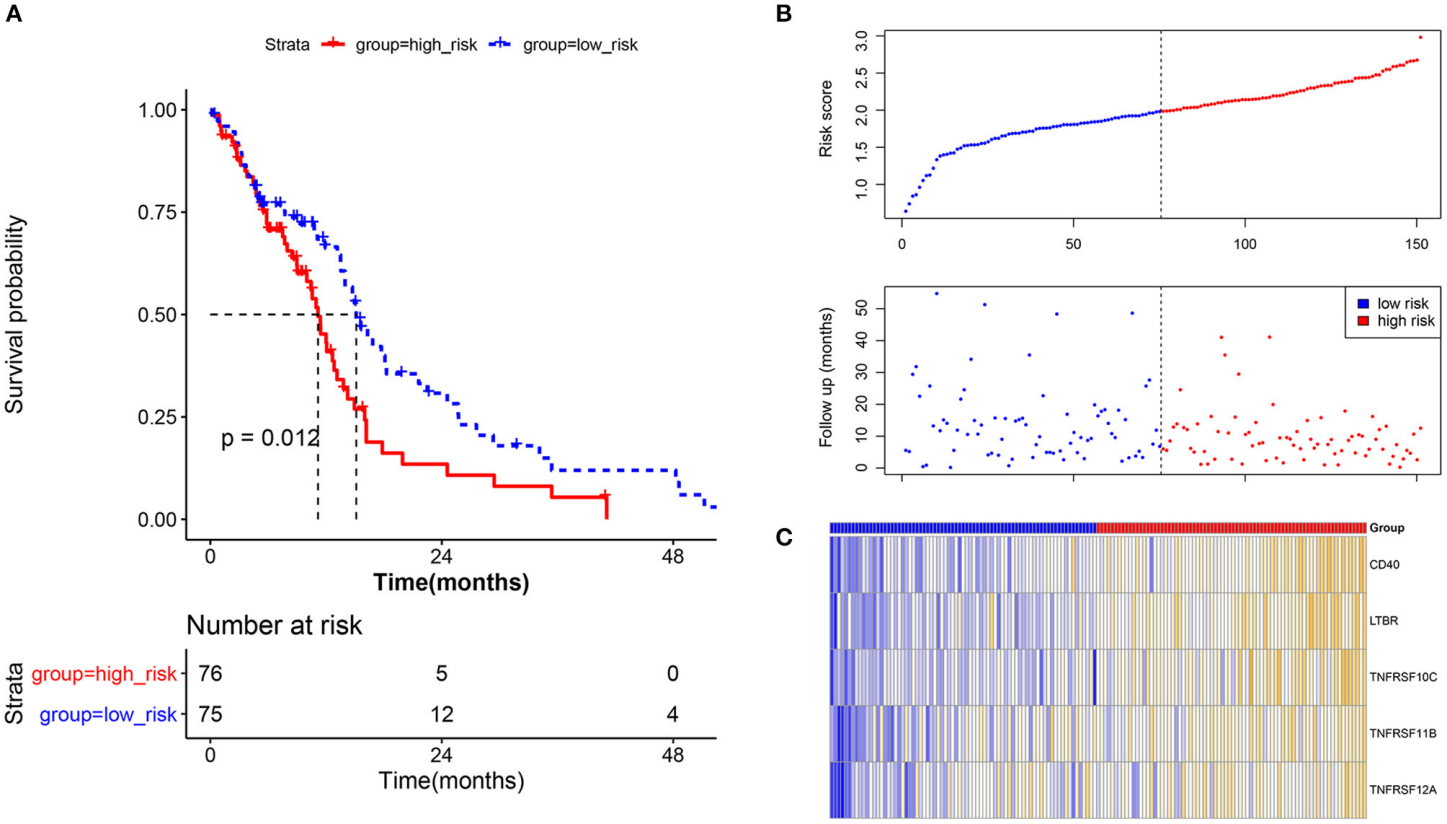
Prognostic verification analysis for the current prognostic model based on tumor samples in the TCGA database. **(A)** Survival analysis for the high-risk group and low-risk group; survival time of the high-risk group was shorter than that of the low-risk group. The X-axis represents the overall survival time (month), while the Y-axis represents the survival rate (percent survival). *P* < 0.05 was considered to be significantly different. **(B)** The risk score and follow-up in the high-risk group and low-risk group. **(C)** The heat map for methylation site-regulated prognosis-related genes including CD40, LTBR, TNFRSF10C, TNFRSF11B, and TNFRSF12A.

A total of 5 methylation site-regulated prognostic genes including CD40, lymphotoxin beta receptor (LTBR), TNF receptor superfamily member (TNFRSF) 10C, TNFRSF 11B, and TNFRSF12A (Supplementary Figure 1) were revealed. Multivariate Cox regression was performed on these 5 candidate genes, followed by the risk model construction. The results showed that the survival time of the high-risk group was shorter than that of the low-risk group (Figure 3A). With the increase of the risk score, the expression level of these 5 candidate genes was relatively higher, and the survival rate of the high-risk group was significantly lower than that of the low-risk group (Figure 3B). The heat map of these 5 candidate genes in each sample is shown in Figure 3C. Moreover, the GBM samples in the CGGA database were used to evaluate the above risk model. The results showed that the prognosis effect of the risk model on the CGGA database were the same with that on the TCGA database (Supplementary Figure 2), which further indicated that the prognosis model constructed by these 5 candidate genes was effective.

### Independence Analysis of the Prognosis Model

**Figure 4:**
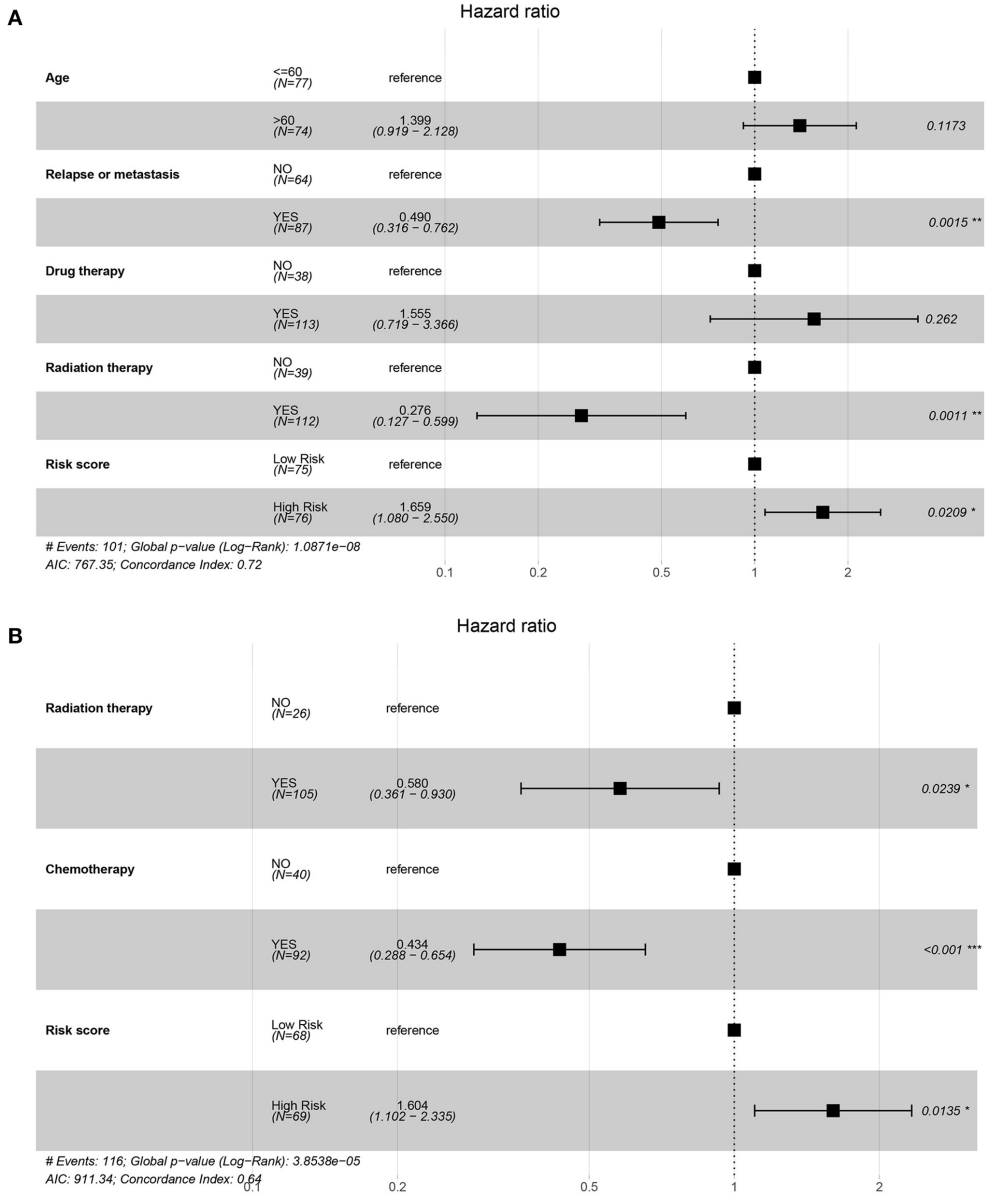
Forest map of regression analysis based on the TCGA and CGGA datasets. **(A)** The forest plot for multivariate Cox regression of the TCGA dataset on factors including age, relapse or metastasis, drug therapy, radiation therapy, and risk score. **(B)** The forest plot for multivariate Cox regression of the CGGA dataset on factors including radiation therapy, chemotherapy, and risk score.

In order to investigate whether the prognosis model could be independent of other clinical variables, the univariate and multivariate Cox regression analyses based on the TCGA dataset and CGGA dataset were performed. In the TCGA cohort, on univariate Cox regression analysis of clinical valuables and risk score, the results showed that age, relapse or metastasis, drug therapy, radiation therapy, and risk score had a statistically significant impact (*P* < 0.05). These valuables were further included in multivariate analysis; the results showed that relapse or metastasis (HR 0.49, 95% CI 0.316–0.762, *P* = 0.002), radiation therapy (HR 0.276, 95% CI 0.127–0.599, *P* = 0.001), and risk score (HR 1.659, 95% CI 1.080–2.550, *P* = 0.021) were variables that independently affect survival (Table 1, Figure 4A).

**Table 1:** The univariate and multivariate Cox regression analysis results for the TCGA dataset.

**Variables**	**Univariate analysis**			**Multivariate analysis**		
	**HR**	**95% CI**	***P* value**	**HR**	**95% CI**	***P-*value**
**Gender**						
Male/female	0.792	0.529–1.184	0.255			
**Race**						
Black race/yellow race	2.361	0.428–13.019	0.324			
White race/yellow race	1.862	0.458–7.580	0.068			
**Age**						
>60/≤60	1.745	1.171–2.600	**0.006**	1.399	0.919–2.128	0.117
**Relapse or metastasis**						
Yes/no	0.439	0.292–0.660	** <0.001**	0.49	0.316–0.762	**0.002**
**Drug therapy**						
Yes/no	0.415	0.271–0.635	** <0.001**	1.555	0.719–3.366	0.262
**Radiation therapy**						
Yes/no	0.307	0.202–0.468	** <0.001**	0.276	0.127–0.599	**0.001**
**Risk score**						
High/low	1.67	1.115–2.501	**0.013**	1.659	1.080–2.550	**0.021**

*HR, hazard ratio; CI, confidence intervals. P < 0.05 was considered to be significantly different*.

In the CGGA cohort, on univariate Cox regression analysis of clinical valuables and risk score, the results showed that radiation therapy, chemotherapy, and risk score had a statistically significant impact (*P* < 0.05). These valuables were further included in multivariate analysis; the results showed that radiation therapy (HR 0.580, 95% CI 0.361–0.930, *P* = 0.024), chemotherapy (HR 0.434, 95% CI 0.288–0.654, *P* < 0.001), and risk score (HR 1.604, 95% CI 1.102–2.335, *P*=0.014) were variables that independently affect survival (Table 2, Figure 4B).

**Table 2:** The univariate and multivariate Cox regression analysis results for the CGGA dataset.

**Variables**	**Univariate analysis**			**Multivariate analysis**		
	**HR**	**95% CI**	***P-*value**	**HR**	**95% CI**	***P-*value**
**Gender**						
Male/female	1.296	0.890–1.886	0.177			
Age						
>60/≤60	1.284	0.733–2.250	0.381			
**Radiation_therapy**						
Yes/no	0.604	0.379–0.965	**0.035**	0.580	0.361–0.930	**0.024**
**Chemotherapy**						
Yes/no	0.475	0.320–0.705	**<0.001**	0.434	0.288–0.654	**<0.001**
**IDH**						
Mutant/wild	0.936	0.634–1.381	0.739			
**1p/19q deletion**						
Co-deletion/non-co-deletion	0.800	0.371–1.727	0.570			
**Risk score**						
High/low	1.438	1.005–2.060	**0.047**	1.604	1.102–2.335	**0.014**

*IDH, isocitrate dehydrogenase; HR, hazard ratio; CI, confidence intervals. P < 0.05 was considered to be significantly different*.

Moreover, the univariate and multivariate Cox regression analyses based on the TCGA dataset showed that radiation_therapy, Chemo_status, and RiskGroup were potential clinical variables that independently affect survival (Table 2). The forest plot for multivariate Cox regression is shown in Figure 4B.

### Protein-Level Verification of Genes in the Prognosis Model

**Figure 5:**
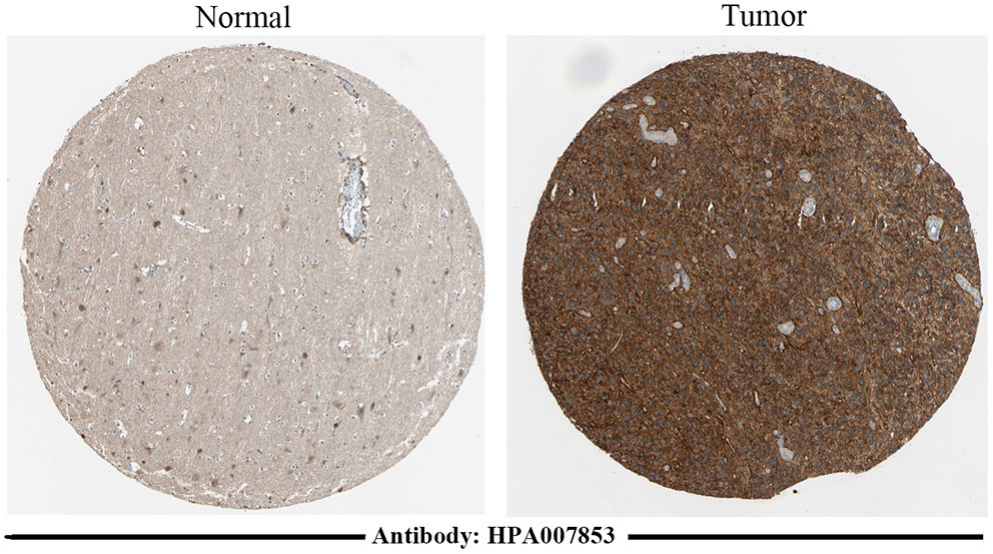
Immunohistochemical staining results of TNFRSF12A based on the HPA database. Immunohistochemical staining of TNFRSF12A in normal tissue [male, age 45; cerebral cortex (T-X2020), NOS (M-00100)] and in tumor tissue [male, age 47; brain (T-X2000) glioma, malignant, high grade (M-938033)].

The protein-level verification based on the HPA database showed that the upregulation and downregulation of proteins between normal and tumor samples were consistent with the expression of TNFRSF12A (Figure 5).

### Drug Prediction Analysis

**Figure 6:**
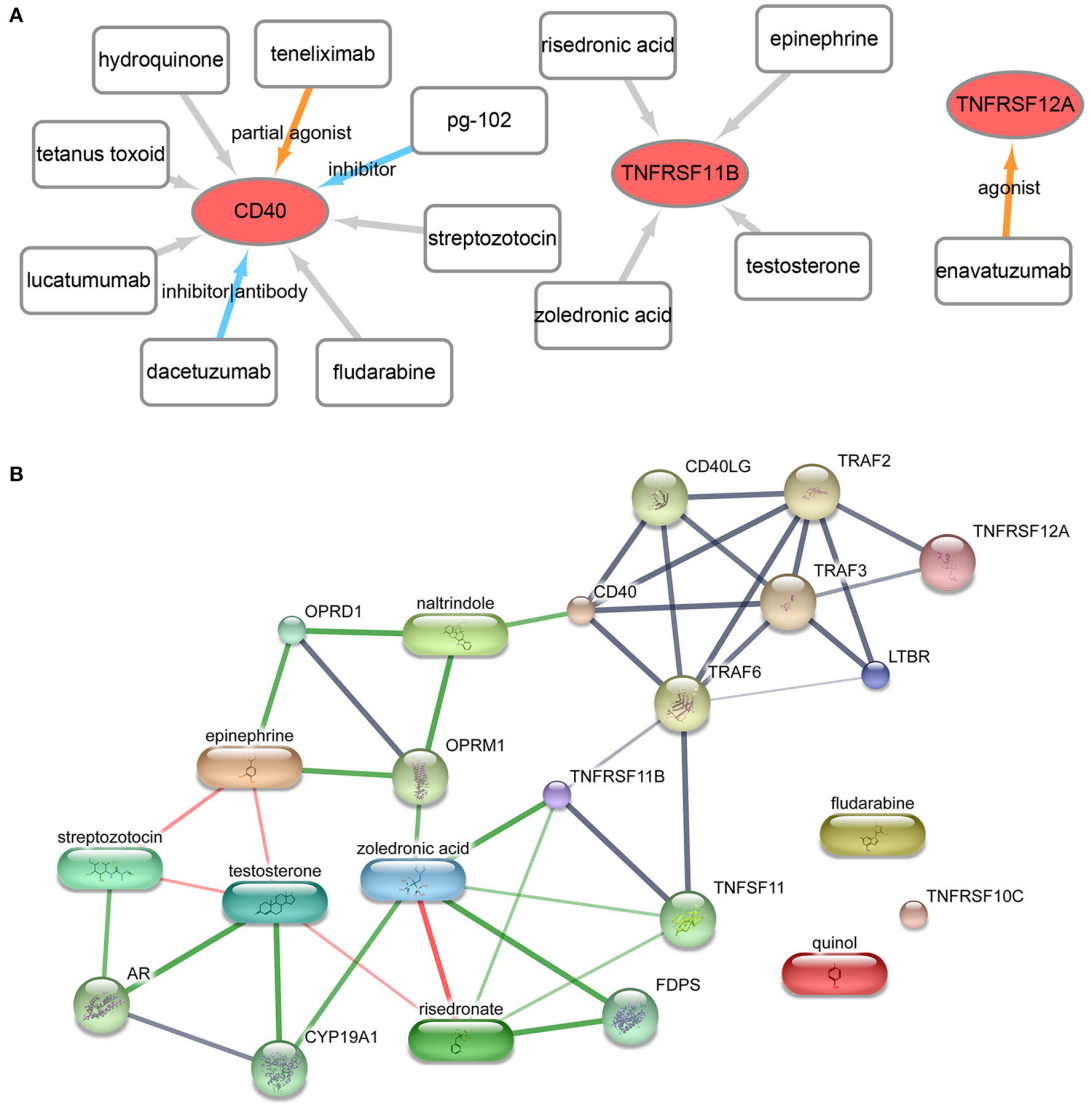
Drug- gene interaction network. **(A)** The drug–gene interaction network from the DGIdb database. Red ellipses represent genes, and rectangles represent drugs; the lines represent drug–gene interactions, of which colored lines represent known interactions while gray lines represent unknown interactions. **(B)** The drug–gene–gene interaction network from the STITCH database. The block represents the drug, while the circle represents gene. Red lines represent the drug–drug interaction, and green lines represent drug–gene interactions, and gray lines represent gene–gene interaction.

A total of 13 gene–drug interaction, including three genes (TNFRSF12A, CD40, and TNFRSF11B) and 13 drug molecules (enavatuzumab, dacetuzumab, pg-102 inhibitor, teneliximab, tetanus toxoid, hydroquinone, streptozotocin, fludarabine, lucatumumab, zoledronic acid, risedronic acid, epinephrine, and testosterone), were explored based on the online database DGIdb (Figure 6A, Supplementary Table 3). Furthermore, the online database STITCH was further used to verify the relationship between drugs and corresponding proteins in genes (Figure 6B, Supplementary Table 4). The result showed that zoledronic acid–TNFRSF11B was the common interaction in both DGIdb database and STITCH database.

## Discussion

GBM is the most common malignant brain tumor in adults (34). Although TNF and methylation sites are proved to be associated with the progression of disease (35), the detailed mechanisms are still unclear. In this study, the bioinformatics analysis showed that there were 41 DEGs between two groups. These DEGs were mainly enriched in GO functions, like response to tumor necrosis factor (GO: 0034612), and in KEGG pathways, like the hsa04668~TNF signaling pathway. Moreover, a total of 5 methylation site-regulated prognosis-related genes including TNFRSF12A, CD40, TNFRSF11B, TNFRSF10C, and LTBR were explored. The prognostic model constructed by the five genes was highly correlated with prognosis both in the TCGA cohort and in the CGGA cohort, and the higher risk score indicated lower survival. Finally, zoledronic acid–TNFRSF11B was revealed as key point drug–gene interaction by drug prediction analysis.

It is widely known that the response to TNF signaling plays an important role during peripheral organ inflammation in the brain (36). This signaling is widely proved to participate in the development of various diseases like ovarian cancer and lung cancer (37, 38). The response to TNF signaling mediates primary resistance to epidermal growth factor receptor inhibition in GBM (39). A previous study shows that TNF-α induces angiogenic factor upregulation in malignant glioma cells which play a role in RNA stabilization during GBM (40). Actually, the biological function of response to TNF is commonly realized by certain genes. TNFRSF12A is the sole signaling receptor for the proinflammatory cytokine TWEAK (TNFSF12) (41). Via TNF signaling such as TWEAK–TNFRSF12A, TNFRSF12A can regulate cellular activities including proliferation, migration, differentiation, apoptosis, angiogenesis, tissue remodeling, and inflammation (41). It has been proved that TNFRSF12A, which plays a role in tumor growth and metastasis, is highly expressed in solid tumor types (42). Yang et al. showed that the upregulation of TNFRSF12A contributed to poor prognosis in cancer (43). A previous study shows that differential expression of TNFRSF12A and DNA methylation contributes to the development of brain diseases such as epilepsy (44). Wang et al. proved that there was a close relationship between TNFRSF12A methylation and carcinoma prognosis (45). Based on the TCGA RNA-sequencing and methylation data, a previous study indicates that the methylation and expression levels of TNFRSF12A is significantly associated with prognosis of hepatocellular carcinoma, which can be used as a prognostic risk model (46). In the current study, TNFRSF12A was one of the five methylation site-regulated prognosis-related genes. Meanwhile, the enrichment analysis showed that TNFRSF12A was one of the genes that assembled in response to TNF function. Importantly, the protein-level verification analysis showed that the upregulation and downregulation of proteins between normal and tumor samples were consistent with the expression of TNFRSF12A. Thus, we speculated that methylation-driven gene TNFRSF12A might take part in the progression of GBM through response to the tumor necrosis factor biological process and TNF signaling pathway and significantly associated with the prognosis of GBM.

Zoledronic acid (ZA) is a potent inhibitor currently used in the clinical treatment of cancer (47). A previous study shows that ZA enhances T-lymphocyte antitumor response to human GBM cell lines (48). The antitumor effect of ZA combined with temozolomide can be used to against human GBM cell DNA methyltransferase (49). Actually, the drug effect of ZA is realized via stimulating the expression of certain genes in GBM cells (50). Karabulut et al. indicated that an induction in mRNA levels of TNFRSF family genes was observed in tumor cells under ZA treatment (51). Genetically achieved disturbances to the expression levels of TNFSF11B can modulate the effects of ZA on growing mouse skeletons (52). TNFRSF11B is a potential plasma biomarker for the clinical diagnosis of various cancers (53, 54). TNFRSF11B is proved to be differentially expressed in many immune cells in the brain (55). GBM is resistant to TNF-receptor family gene-induced apoptosis (56). A previous study indicates that the expression of TNF-receptor family genes including CD70 and TNFRSF11B was associated with the progression of GBM (57). In this study, the drug prediction analysis ZA–TNFRSF11B was revealed as the unique drug–gene interaction in both databases. Meanwhile, TNFRSF11B was revealed as one of the five GBM candidate genes in the methylation site-regulated prognosis-related gene analysis. Thus, we speculated that ZA targeting TNFRSF11B expression might be a potentially effective drug for GBM clinical treatment. However, there were some limitations in this study. Firstly, we preliminarily constructed a five-gene prognosis model. The prognostic performance of the five-gene prognosis model should be further confirmed by clinical samples. Secondly, we identified several potential drug targets. The drug–gene interactions should be validated by experiments, and the clinical value should be further investigated.

In conclusion, the methylation-driven gene TNFRSF12A might take part in the progression of GBM through response to the tumor necrosis factor biological process and TNF signaling pathway and significantly be associated with the prognosis of GBM. Moreover, ZA targeting TNFRSF11B expression might be a potentially effective drug for GBM clinical treatment.

## Data Availability Statement

The datasets presented in this study can be found in online repositories. The names of the repository/repositories and accession number(s) can be found in the article/Supplementary Material.

## Author Contributions

W-cL conceived and designed this research. HX carried out the plan and wrote this paper. CY, J-jL, and Z-yL gave advice and carried out the data analysis. All authors read and approved the final manuscript.

## Conflict of Interest

The authors declare that the research was conducted in the absence of any commercial or financial relationships that could be construed as a potential conflict of interest.

## Supplementary Material

Supplementary Figure 1 The Venn diagram for integrate analysis based on prognostic genes, prognostic methylation, and genes negatively related to methylation.

Supplementary Figure 2 Prognostic verification analysis for current prognostic model based on tumor samples in CGGA database. **(A)** Survival analysis for high-risk group and low-risk group; survival time of high-risk group was shorter than that of low-risk group; The X-axis represented the overall survival time (month), while the Y-axis represented the survival rate (percent survival). *P* < 0.05 was considered to be significant different. **(B)** The risk score and follow-up in high-risk group and low-risk group. **(C)** The heatmap for methylation site regulated prognosis-related genes including CD40, LTBR, TNFRSF10C, TNFRSF11B, and TNFRSF12A.

Supplementary Table 1 The detailed information of the 76 TNF superfamily-related genes.

Supplementary Table 2 The 74 methylation sites were correlated with prognosis.

Supplementary Table 3 The obtained drug-gene interactions from DGIdb database.

Supplementary Table 4 Validation of interactions among the obtained drugs and genes in STITCH database.
