# Supplementary figures and images for: Potential Molecular Mechanism of TNF Superfamily-Related Genes in Glioblastoma Multiforme Based on Transcriptome and Epigenome
Source: Front Neurol. 2021 Feb 11;12:576382. doi: 10.3389/fneur.2021.576382 (PMC7905170; doi:10.3389/fneur.2021.576382)

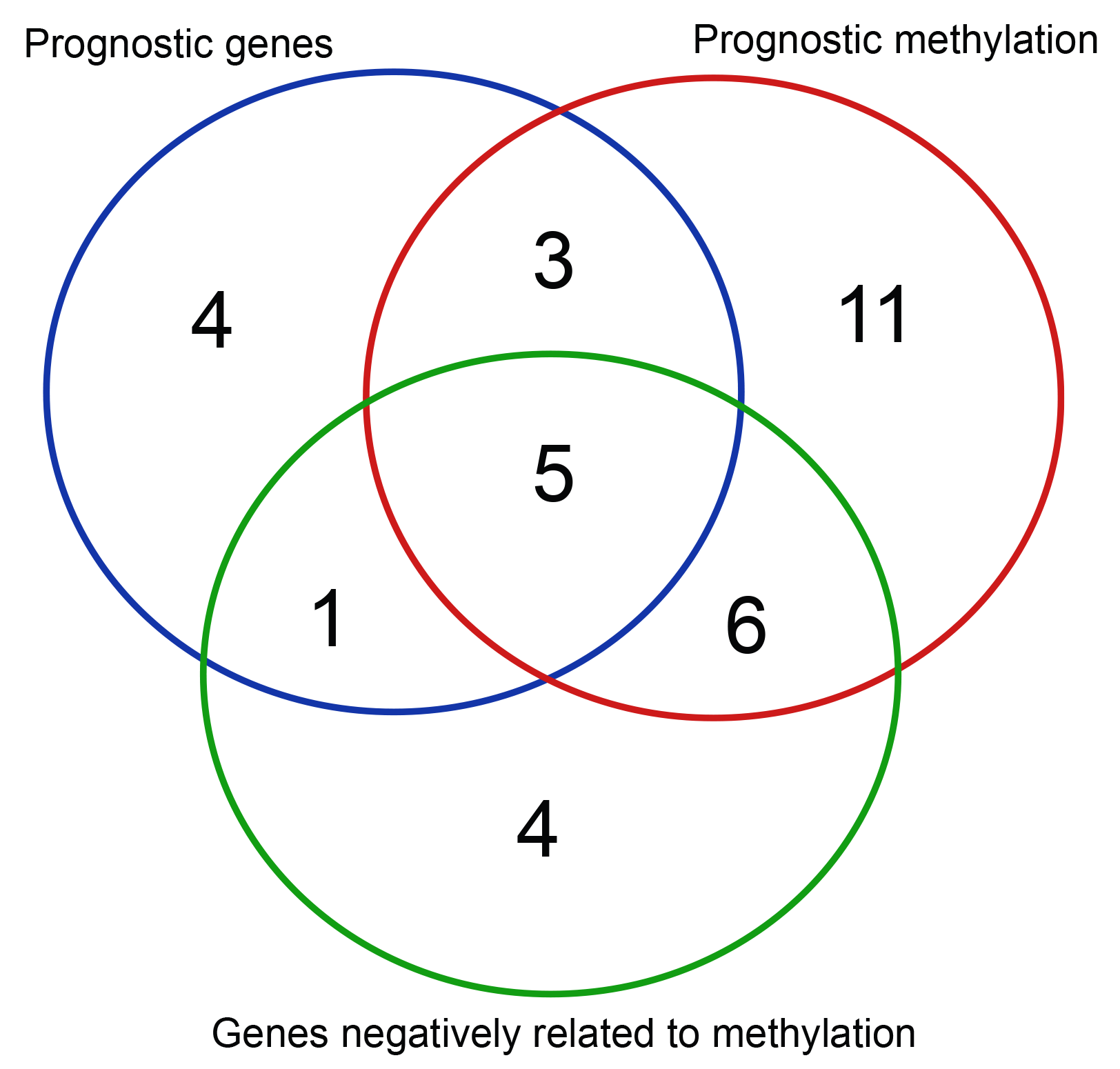

Supplement: Supplementary Figure 1 — The Venn diagram for integrate analysis based on prognostic genes, prognostic methylation, and genes negatively related to methylation. [file Image_1.TIF]

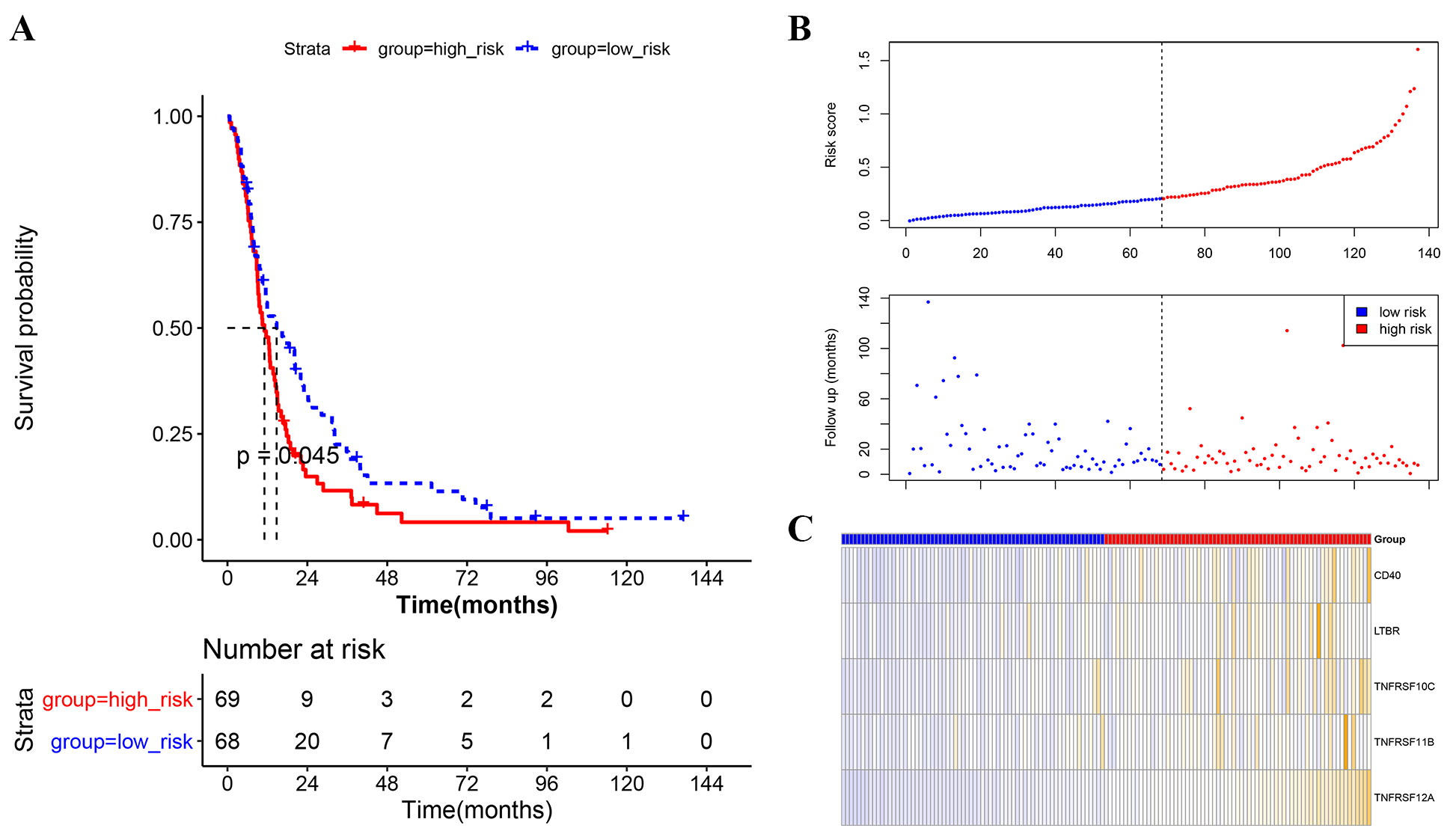

Supplement: Supplementary Figure 2 — Prognostic verification analysis for current prognostic model based on tumor samples in CGGA database. (A) Survival analysis for high-risk group and low-risk group; survival time of high-risk group was shorter than that of low-risk group; The X-axis represented the overall survival time (month), while the Y-axis represented the survival rate (percent survival). P < 0.05 was considered to be significant different. (B) The risk score and follow-up in high-risk group and low-risk group. (C) The heatmap for methylation site regulated prognosis-related genes including CD40, LTBR, TNFRSF10C, TNFRSF11B, and TNFRSF12A. [file Image_2.TIF]
